# Therapeutic Uses of Bacterial Subunit Toxins

**DOI:** 10.3390/toxins13060378

**Published:** 2021-05-26

**Authors:** Clifford Lingwood

**Affiliations:** 1 Division of Molecular Medicine, Research Institute, Hospital for Sick Children, Toronto, ON M5G 1X8, Canada; cling@sickkids.ca; 2 Departments of Laboratory Medicine & Pathobiology, and Biochemistry, University of Toronto, Toronto, ON M5S 1A8, Canada

**Keywords:** retrograde transport, neoplastic Gb_3_ expression, endoplasmic reticulum associated degradation, protein misfolding diseases

## Abstract

The B subunit pentamer verotoxin (VT aka Shiga toxin-Stx) binding to its cellular glycosphingolipid (GSL) receptor, globotriaosyl ceramide (Gb_3_) mediates internalization and the subsequent receptor mediated retrograde intracellular traffic of the AB5 subunit holotoxin to the endoplasmic reticulum. Subunit separation and cytosolic A subunit transit via the ER retrotranslocon as a misfolded protein mimic, then inhibits protein synthesis to kill cells, which can cause hemolytic uremic syndrome clinically. This represents one of the most studied systems of prokaryotic hijacking of eukaryotic biology. Similarly, the interaction of cholera AB5 toxin with its GSL receptor, GM1 ganglioside, is the key component of the gastrointestinal pathogenesis of cholera and follows the same retrograde transport pathway for A subunit cytosol access. Although both VT and CT are the cause of major pathology worldwide, the toxin–receptor interaction is itself being manipulated to generate new approaches to control, rather than cause, disease. This arena comprises two areas: anti neoplasia, and protein misfolding diseases. CT/CTB subunit immunomodulatory function and anti-cancer toxin immunoconjugates will not be considered here. In the verotoxin case, it is clear that Gb_3_ (and VT targeting) is upregulated in many human cancers and that there is a relationship between GSL expression and cancer drug resistance. While both verotoxin and cholera toxin similarly hijack the intracellular ERAD quality control system of nascent protein folding, the more widespread cell expression of GM1 makes cholera the toxin of choice as the means to more widely utilise ERAD targeting to ameliorate genetic diseases of protein misfolding. Gb_3_ is primarily expressed in human renal tissue. Glomerular endothelial cells are the primary VT target but Gb_3_ is expressed in other endothelial beds, notably brain endothelial cells which can mediate the encephalopathy primarily associated with VT2-producing *E. coli* infection. The Gb_3_ levels can be regulated by cytokines released during EHEC infection, which complicate pathogenesis. Significantly Gb_3_ is upregulated in the neovasculature of many tumours, irrespective of tumour Gb_3_ status. Gb_3_ is markedly increased in pancreatic, ovarian, breast, testicular, renal, astrocytic, gastric, colorectal, cervical, sarcoma and meningeal cancer relative to the normal tissue. VT has been shown to be effective in mouse xenograft models of renal, astrocytoma, ovarian, colorectal, meningioma, and breast cancer. These studies are herein reviewed. Both CT and VT (and several other bacterial toxins) access the cell cytosol via cell surface ->ER transport. Once in the ER they interface with the protein folding homeostatic quality control pathway of the cell -ERAD, (ER associated degradation), which ensures that only correctly folded nascent proteins are allowed to progress to their cellular destinations. Misfolded proteins are translocated through the ER membrane and degraded by cytosolic proteosome. VT and CT A subunits have a C terminal misfolded protein mimic sequence to hijack this transporter to enter the cytosol. This interface between exogenous toxin and genetically encoded endogenous mutant misfolded proteins, provides a new therapeutic basis for the treatment of such genetic diseases, e.g., Cystic fibrosis, Gaucher disease, Krabbe disease, Fabry disease, Tay-Sachs disease and many more. Studies showing the efficacy of this approach in animal models of such diseases are presented.

## 1. Introduction

The Verocytotoxin (VT, Shiga toxin: Stx) was first shown to be the cause of the hemolytic uremic syndrome (HUS) in 1985 by Karmali [1]. HUS was primarily a potentially fatal acute pediatric renal disease of unknown origin with a triad of symptoms: renal glomerular infarct, thrombocytopenia and anemia [2]. VT is a family of A-B5 subunit *E. coli* toxins [3] in which VT1 and VT2 are primarily associated with HUS [4,5]. Gastrointestinal infection with VT producing *E. coli* initially causes bloody diarrhea and the resulting systemic toxemia leads to renal disease some days later. VT2 is more commonly associated with human disease [6,7] and is additionally associated with encephalopathy [8].

The receptor of the VT B subunit pentamer is the glycosphingolipid, globotriaosyl ceramide, Gb_3_: Galα 1-4 galβ1-4Glc-cer [9]. This is the only VT receptor to mediate cytopathology [10,11]. VT1 shows a higher Gb_3_ binding affinity than VT2 [12]. Gb_3_ is highly expressed in human renal glomerular endothelial cells [13]. Membrane GSLs, together with cholesterol, are major components of lipid rafts which are membrane areas of increased ‘order’ [14] which serve as foci of transmembrane signalling [15] and portals for microbial–host cell interactions [16,17]. VT bound Gb_3_ is internalized by clathrin-dependent [18,19] and independent [20,21,22] mechanisms. Pentameric VT binding can cluster cell surface Gb_3_ to induce membrane curvature, invagination and vesicle scission [21,22,23]. Internalized VT is trafficked to endosomes and thence, via retrograde transport, to the Golgi and then ER. VT-> ER targeting is required for pathogenesis and requires that the cell surface bound Gb_3_ is within lipid rafts [24]. Non-raft Gb_3_ bound VT is, in contrast, internalized and transported to lysosomes [24]. This sorting corresponds with the presence of Gb_3_ in rafts in human renal glomerular endothelial cells, the site of primary pathology [25], but not in renal tubular epithelial cells [13] (which are affected only in later HUS stages [13]). The microvascular endothelial cell raft and non-raft Gb_3_ fatty acid composition has recently been found to vary, with saturated species restricted to the raft and unsaturated species to the non-raft fraction [26]. Moreover, different Gb_3_ fatty acid binding preferences of distinct Gb_3_ binding ligands can result in differential raft membrane Gb_3_ clustering [27].

In the ER, the A and B subunits separate [28] and a C-terminal A subunit sequence, which mimics an unfolded protein [29] and can insert in the ER membrane [30], becomes exposed. This ‘misfolded peptide’ recruits the ERAD (host cell quality control ER associated degradation) machinery [31] to transit the A subunit through the ER membrane via the ERAD dislocon [32] to the cytosol. Here, the A subunit avoids proteasomal degradation by mechanisms which prevent lysine ubiquitinylation [31] and inhibits protein synthesis via an RNA N-glycanase activity [33]. A similar trafficking pathway is followed by cholera toxin A subunit [34]. CT binds the widely expressed GSL, GM1 ganglioside, and similarly undergoes (raft dependent [35]) retrograde transport to the ER [36]. The cytosolic transit requires the chaperone-mediated unfolding of the A subunit and the threading of the linearized subunit through the membrane dislocon (retrotranslocon) to the cytosol where the A subunit refolds. This is unlike natural misfolded proteins which are unfolded in the ER and retrotranslocated through the dislocon via ubiqitinylation, to the cytosolic proteosome for degradation [37]. The VT and CT A subunits escape degradation by incompletely understood mechanisms which may be lipid-raft-dependent [31], and refold in the cytoplasm to effect cytopathology.

The exact composition and ATP dependent action mechanism of the dislocon are still a matter of debate [31,38] and varies as a function of substrate. Reverse transit of the Sec61 translocon was initially considered the primary mechanism [39]. While Sec61 retains a central role in current translocation models [31,40,41,42], more recent studies show more complex mechanisms. The Derlin proteins [43,44], p97 ATPase [45], Hrd 1 ubiquitin ligase [46,47], and SEL1 adaptor protein [48] are established components (37). Recently, Hrd 1 alone has been shown—as predicted [46]—to form pores in model membranes [49]. Both VT [50] and CT hijack the dislocon [51] and indeed, CT has been frequently used a tool to investigate ERAD [31,52,53].

In addition to inhibition of protein synthesis, VTs have been shown to induce apoptosis [54,55,56,57,58,59,60,61,62]. This can be due to the pentameric B subunit binding Gb_3_ [63,64,65], a property shared by anti-Gb_3_ [66], or require the intact holotoxin [62,67]. VT1 induces apoptosis in many human cancer cell lines [68,69,70,71,72], but VT-induced ER stress can also activate survival pathways [73].

## 2. Gb_3_ Is a Cancer Marker

Prior to the discovery of the VT receptor role for Gb_3_, Gb_3_ was considered only a minor GSL in biosynthesis of the major red cell GSL, globoside, Gb_4_ [74]. Gb_4_ is part of the P blood group in which Gb_3_ is Pk [75]. Its sole claim to fame was that of being the BLA-Burkitt lymphoma antigen [76,77]. Gb_3_ was shown to be CD77, a germinal center B cell antigen [78] required for B cell apoptosis [79]. However, even at these early stages, Gb_3_ became associated with lymphoid malignancy [80].

Gb_3_ is upregulated in many human cancers [81,82]. These include breast [72,83], ovarian [84,85], Burkitt’s lymphoma [63], hairy cell leukemia [86], megakaryoblastic leukemia [87], myeloma [88], post-transplant lymphoproliferative disease [89], renal [90], colorectal [65,91], gastric [70,92], testicular [90,93], prostate [90], pancreatic [94,95,96], sarcoma [97], glioma [71], astrocytoma [98,99] and meningioma [100].

Importantly, irrespective of the primary tumour Gb_3_ expression status, the proliferating endothelial cells of the tumour neovasculature also express Gb_3_, [101], and are, therefore, also targeted by VT in tumour xenograft models [100,102,103], to block tumour angiogenesis. VT binding has been proposed as a marker of tumour neovasculature [104] and tumour infiltrating blood vessels [81]. Anti Gb_3_ also prevents neoangiogenesis and tumour growth [105,106].

VT has been shown effective to prevent the growth of many Gb_3_ expressing human tumour xenografts in mice [65,70,90,98,100,104]. In these models, targeting of the neovasculature, in addition to the tumour per se, was observed [98,100,101]. In colon cancer, expression of Gb_3_ synthase was found to be sufficient to initiate metastases [65] and Gb_3_ expression correlates with lymphoma tumorigenicity [107].

### 2.1. Is This the Limit of Gb_3_ Detection?

Although many tumours express Gb_3_, this may represent an underestimate of potential tumour targeting. The receptor function of membrane Gb_3_ (and other GSLs) is complex [108] in that the lipid moiety [26,109] and the membrane environment [26,110] play a central role in Gb_3_ accessibility for ligand binding GSL. Differential tissue binding of VT and anti-Gb_3_ antibodies [111] and the differential detection of cell surface Gb_3_ by VTB and various antiGb_3_ Mabs [112] further indicate the importance of Gb_3_ membrane presentation. This has been termed cryptic GSL expression [113]. The carbohydrate moiety of GSLs can exist in a variety of conformations and this is affected by the GSL lipid moiety and relative plane of the membrane [114]. While the exact basis of this is not known in all cases, one situation has been mechanistically characterized. GSLs and cholesterol are the major component of lipid rafts [115]. In the membrane GSL-cholesterol complex, H-bonding results in a change in the GSL sugar conformation, from a membrane perpendicular to membrane parallel format [116,117]. In the parallel format, access for exogenous ligand binding is severely restricted. We have used the term ‘invisible GSLs’ to describe this phenomenon [118]. β-Methyl cyclodextrin (MCD) has been used to selectively extract cholesterol from cells in vitro [119] and in vivo [120]. Treatment of human tumour sections with MCD can markedly increase tumour GSL ‘exposure’ and the binding of VT1 (and other tumour associated GSL binding ligands) is greatly increased [121]. Less than 10% of tumour Gb_3_ may be available for VT binding without prior cholesterol extraction. Thus, MCD markedly increases membrane GSL exposure.

However, the fact that Gb_3_ needs to be within (cholesterol containing?) lipid rafts for VT cell killing [24] needs to be considered. A balance between cholesterol extraction for increased binding and potentially reduced ER trafficking needs to be achieved.

### 2.2. Cancer Stem Cells

Glycosphingolipid antigens have been used as markers of human stem cells [122,123,124] and cancer stem cells [125,126]. These are, for the most part, globoseries GSLs, and Gb_3_ has been defined as a marker of breast cancer, but not normal mammary, stem cells [127]. However, this has been contested [128]. Moreover, cancer stem cell express high levels of ABC transporters [129], which can be involved in GSL biosynthesis [130].

## 3. Verotoxin as a Cancer Targeting Tool

The extremely high binding affinity of the B subunit pentamer for membrane Gb_3_ (>10^−9^ M [131,132]) and the widespread overexpression of Gb_3_ in human cancers makes verotoxin an attractive antineoplastic targeting tool.

### 3.1. B Subunit Conjugates

Due to the concern that VT holotoxin is central in the etiology of HUS, several anti-proliferative drugs have been conjugated to the nontoxic VT B subunit to increase tumour selective cytotoxicity [92,133,134,135]. This has been combined with delivery of a prodrug which requires subsequent activation [133,134], since, if side effects are a problem for the therapeutic use of the holotoxin, changing the toxic moiety delivered should not affect targeting and or side effects. In addition, B subunit conjugates have been developed to provide tools for in vivo tumour imaging [83,91,101,103]. Fluorescent VT/VTB has long been used to image Gb_3_ positive cells in vitro [20,136]. B subunit conjugates containing additional C terminal peptides retain native Gb_3_ binding [137]. The addition of a C terminal cysteine residue to the B subunit has facilitated the generation of such conjugates via disulfide linkage [138]. Such linker conjugates will be cleaved by reduction in the tumour ER [135,139,140] to allow specific ER cargo delivery [141]. A peptide which promotes ER retrotranslocon transit can be coupled to increase cytosolic cargo access [142,143].

In a different approach, Lactococcus bacteria have been transfected to express surface VTB as a potential antineoplastic drug carrier [144].

In addition, a diphtheria toxin A subunit-VTB subunit conjugate was modeled [145] as a novel antineoplastic [145]. While it appears counterintuitive to couple a less efficacious A subunit to VTB, concern about potential VT holotoxin side effects is still evident.

### 3.2. Native VT1 in Cancer Therapy

#### 3.2.1. Potency

The use of the native VT1 toxin provides a far more potent antineoplastic approach than coupling drugs to the B subunit. Although the Gb_3_ binding affinity is in the nanomolar range, VT1 has proven cytotoxic in vitro at a dose of 10^−13^–10^−17^ M, [146,147], up to eight orders of magnitude lower. It has been proposed, and consistent evidence reported [148], that the catalytic activity of one cytosolic A subunit molecule is sufficient to kill a cell.

#### 3.2.2. Risk

The question is ‘does the antineoplastic use of the VT holotoxin pose a risk for HUS?’. To address this question, firstly the demographics of VTEC HUS need to be considered. HUS is primarily a disease of the young and the elderly [149]. Thus, there is an age window. Secondly, pathology is more closely associated with VT2 producing EHEC than VT1 [150,151]. Thirdly, can VT1 alone cause HUS? In this regard, the baboon is the only appropriate model. In the baboon primate model, an i.v. bolus dose of 100 ng/kg VT1 was sufficient to induce the classical symptoms of HUS [152,153]. However, if this same VT1 dose was divided into four which were administered every 12 h for 48 h, no subsequent symptoms of HUS were observed [153]. At equal doses, only VT2 induced pathology [154]. Thus, there is a ‘safe’ sterile VT1 i.v dose which can be repeated for four days (at least).

VT1 antineoplastic treatment may temporarily dysregulate erythropoiesis [155,156] but if necessary, transfusion is a clear option.

In the in vivo context of gastrointestinal EHEC infection and presumed systemic verotoxemia, the situation is more complex. Bacterial LPS [157,158] and various cytokines induced by the infection [159,160,161,162] can upregulate cellular synthesis of Gb_3_ [163,164,165], increasing VT susceptibility and the number of cells killed by VT [161,166]. Indeed, LPS coadministration increased VT pathology in the baboon [167]. In addition, VT can induce the expression of such cytokines [168,169,170]. Thus, HUS is the long term (3–7 days) culmination of a ‘perfect storm’ arising from the initial gastrointestinal EHEC infection. This provides a therapeutic opportunity for a sterile VT1 bolus. Furthermore, Gb_3_ receptor expression within lipid rafts in normal cells is required for cytopathology and tumour cells have higher levels of lipid rafts [171]. Finally, HUS is rarely fatal whereas cancer, e.g., pancreatic, always is. Any potential circulating VT-induced renal pathology may prove treatable [172,173]

Intratumoural VT1 administration into a Gb_3_ expressing tumour provides a further means to reduce risk. Although circulating VT will concentrate in a Gb_3_ positive tumour [92,103], high affinity receptor binding within the tumour following judicious i.t. administration, will localize the toxin to the tumour and limit systemic access. In human tumour xenograft models, intratumoural VT injection can eliminate the tumour, which indicates the toxin spreads throughout the tumour from the injection site. Derivatized VTB can be used to image Gb_3_ expressing tumours in vivo [101,174] and could thereby serve to accurately guide intratumoural treatment and to monitor response efficacy.

#### 3.2.3. Efficacy

Comparison of the antineoplastic efficacy of B subunit-drug conjugates and native VT1 holotoxin have not been made. However, tumour xenografts were reduced by 50% by an optimized B subunit-p53 inhibitor complex after 6× 10 mg/kg administrations every 48 h [143], whereas a single 4µg/kg VT1 treatment eliminated a xenograft tumour within 7 days [102]. In these first tumour xenograft studies, we determined that a single intratumoural dose of 1 µg VT1/50 mm diameter tumour was sufficient to reduce the astrocytoma size by 50% within 2 days with tumour elimination after about a week [102]

## 4. Verotoxin Interaction with Lymphoid Cells

### 4.1. As an Immunogen Carrier

Since human germinal center B cells [175] and more importantly, dendritic cells [176] express Gb_3_, verotoxin B subunit [138,177] or holotoxin [178] can be used as a carrier to more effectively deliver immunogens to the cytosol for proteasomal processing. Peptides thus generated, can readily transit back to the ER via the TAP peptide transporter [138,179] for loading onto MHC-1 within the ER for anterograde transport and cell surface antigen presentation [176,180,181]. ER antigen targeting facilitates dendritic cell proteasomal antigen processing and is, therefore, a more efficient immunization protocol [182]. This procedure was utilized to develop a cancer vaccine against the Her2/neu breast tumour antigen [181]. VTB subunit is an effective murine nasal mucosa immunogen vector [183,184] to elicit resident memory T cells and antitumour antibodies. Significantly, VT1 was found to bind extensively to mouse nasal turbinates [185] which, if reflective of the human nasal mucosa, would provide a unique immunoantineoplastic carrier.

### 4.2. Effects on Lymphoid Cells

The binding of VT to B lymphocytes is complex. Gb_3_ is also known as CD77 [186], a differentiation antigen expressed on germinal centre B cells [79] associated with apoptosis [187]. Due to immunoglobulin isotype switching in germinal centres [188] and the inhibition of IgG/A production by VT in vitro, we suggested this was a basis for the failure to develop long-lived immunity to VT after infection [189]. Despite this early work, the effect of VT on the immune system remains largely unknown. VT treated monocytes release proinflammatory cytokines [190] which may be enhanced by neutrophil VT delivery [191].

Recent reports show VT can have a surprising caspase-mediated anti-inflammatory activity against LPS [192] in macrophages and in mice. It may, therefore, be possible eventually, to use VT or derivative, as an anti-inflammatory or to identify anti-inflammatory targets. Interestingly, macrophage Gb_3_ is not expressed in lipid rafts [24] and VT retrograde transport to the ER does not occur. This lack of VT susceptibility may permit this (and other? e.g., [193]—see below) A subunit, holotoxin effects.

## 5. Verotoxin A Subunit Redirection of Intracellular Traffic

In a recent interesting and perhaps landmark study [193], the catalytic A subunit inactivated holotoxoid of VT2a(Stx2a) was found to partially undergo a differential intracellular traffic route (as opposed to the retrograde ER transport of all other verotoxin family members) from the cell surface to the lysosome for degradation, consistent with the reported more varied intracellular trafficking of the VT2 toxin family [136]. This lysosomal trafficking pathway is normally followed for VT binding non-raft cell surface Gb_3_ [24], which would suggest that this VT2a toxoid is able to selectively bind non-raft cell surface Gb_3_. In addition, the inactivated VT2a holotoxin was able to redirect the traffic of amyloid precursor protein (APP) to the lysosome for degradation in treated cells also. This intracellular misdirection of APP prevented the subsequent cellular release of the amyloid-β peptide, and thereby may provide a novel basis for the exogenous therapy of Alzheimer’s disease, particularly since VT2 is more frequently associated with the encephalopathogenic features of VT producing *E. coli* infections [8].

The mechanistic basis of such an approach is worthy of consideration. The Gb_3_ binding and subsequent intracellular traffic is B subunit dependent. Why is the (inactivated) A subunit required? Perhaps this is related to VT2 A subunit binding serum amyloid P [194] and the relationship between amyloid P and β amyloid [195,196,197,198]. Retrograde traffic to [24,199,200], and anterograde traffic from [201] the ER, are cholesterol dependent and the interactions between cholesterol and GSLs [202,203,204] can oppositely affect the GSL binding of VT [117] and β amyloid [116,205,206,207]. GSL biosynthesis promotes APP and β amyloid secretion [208] and both membrane GSLs and cholesterol regulate the β secretase which generates β amyloid [209].

## 6. Cholera Toxin as a Targeted ER Associated Degradation (ERAD) Blockade

Both VT and CT hijack endoplasmic reticulum associated degradation (ERAD) for A subunit cytosolic access. ERAD is the cellular quality control mechanism by which nascent polypeptides are screened for correct three dimensional folding [210]. It involves their sampling by various ER chaperones [211,212] and folding enzymes [213], unfolding/refolding opportunities [214], unfolding [215], ubiquitinylation [216], ER-cytosolic transfer [217] and proteasomal degradation [218]. Protein misfolding is central in many genetic diseases, and in those with small mutations that do not ablate function, ERAD plays a key role in initiating/amplifying deficiency disease symptoms [219].

### Intracellular Plumbing

**Figure 1:**
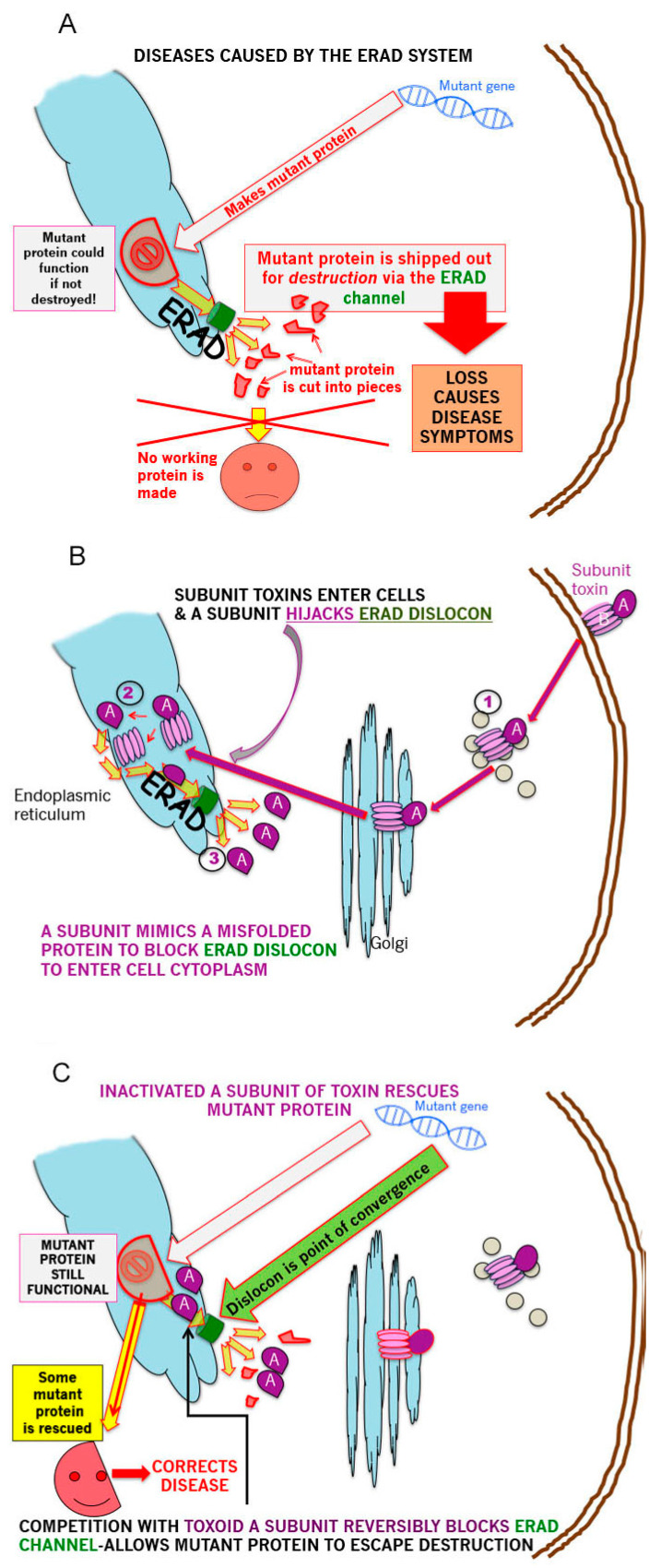
Hijacking the hijacker. (**A**). Genetic diseases in which a small (e.g., point) mutation induces partial protein misfolding while retaining a significant fraction of the wildtype protein function, are candidates for this approach. The misfolded mutant is selected by ER chaperones for degradation by the cytosolic proteosome. Such proteins are unfolded, ubiquinylated and translocated across the ER membrane via the ERAD channel—the dislocon (indicated in green). This cytosolic destruction precipitates/exacerbates the deficiency disease symptoms. (**B**). Several bacterial subunit toxins hijack the ERAD translocon by A subunit mimicry of an unfolded protein. The holotoxin undergoes (GSL-dependent for VT and CT) retrograde from the cell surface to the ER. Subunit separation (via PDI) then allows the A subunit to enter and transit the dislocon which can only transport one polypeptide at a time. (**C**). Thus, the exogenous (inactivated toxin A subunit) and endogenous (mutant misfolded protein) translocation substrates converge at the ER dislocon. A subunit occupancy of the dislocon will compete for mutant protein transit, and thereby allow a fraction of the mutant protein to avoid ERAD and traffic to its correct cellular address to rescue (at least in part) the deficiency disease phenotype.

The GSL mediated retrograde transit of exogenous CT (GM1 ganglioside receptor), (VT or other subunit toxins) to the ER to deliver the A subunit, via the ERAD retrotranslocon, (dislocon) to the cytosol, represents a major opportunity to carry out precisely targeted, intracellular plumbing to stop (slow) the ‘leak’ of mutant partially misfolded proteins into ERAD and thereby ameliorate the symptoms of multiple genetic protein misfolding deficiency diseases this degradation causes. This is represented schematically in the 3 panels of Figure 1—panel A shows the basic problem for ERAD-dependent protein misfolding diseases; panel B, the pathway by which subunit toxins hijack ERAD; panel C shows the means by which this hijacking can be turned to advantage to partially correct disease symptoms.

As long as the mutant protein retains a fraction of the wildtype activity, ‘plugging’ the ER dislocon should result in increased ERAD escape and the delivery of sufficient protein of sufficient function to the final target site, to reverse disease symptoms. Inactivation of the catalytic activity of the A subunit generates a holotoxin which will deliver a benign A subunit to the dislocon which when thus occupied, should be unable to simultaneously translocate misfolded nascent mutant protein to the proteosome for degradation. This is a titratable system and, therefore, up to a limit, it should be possible to ‘dial in’ the required level of mutant protein ERAD escape. It was previously calculated that it took 50 molecules of toxin to accumulate in the ER to ‘generate’ 1 A subunit in the cytosol [148]. This could indicate a slow A subunit translocation rate, which would be beneficial in this scenario, or poor unfolded protein mimicry, which would not. To prolong A subunit dislocon residence time, a C terminal polyleucine ‘stop transfer’ sequence was also added [220]. It should be noted that the use of disease-specific pharmacological chaperones in such misfolding diseases [221,222] is compatible as a combination therapy.

## 7. Treatment for Protein Misfolding Diseases

### 7.1. F508delta CFTR

The F508delta CFTR mutation is the most common cause of cystic fibrosis [223]. This single amino acid deletion results in the partial misfolding and destabilization of the CFTR chloride transporter [224,225] and its removal by ERAD [226]. Even wildtype CFTR itself is partially subject to ERAD [227]. F508delta CFTR retains significant chloride transport activity in vitro [228].

We first demonstrated the efficacy of this novel toxoid approach by treating HeLa cells expressing the F508delta CFTR mutation [220]. In these cells, none of the mature glycosylated CFTR (band c-lactosamine glycosylated) could be detected by Western blot but rather a small fraction of the band b (high mannose glycosylated) immature form was present. Even in the presence of wildtype VT1, when protein synthesis was completely inhibited, increased levels of the mature CFTR were detected after 2 h treatment. VT1 containing an inactivated A subunit was similarly effective. Since Gb_3_ expression is highly tissue selective, we switched to cholera toxin, whose receptor is the widely distributed GM1 ganglioside, in order to develop a generally applicable approach to ERAD exacerbated protein misfolding diseases. A subunit inactivated CT (mCT) was able to promote F508delta CFTR ERAD escape and partially restore cellular CFTR function (chloride transport) [220].

### 7.2. N370S Glucocerebrosidase

In vitro, mCT was also able to rescue cellular mutant glucocerebroside (GBA) N370S [220], the misfolding of which and subsequent ERAD, is the cause of Gaucher Disease [229], in which the glycosphingolipid substrate, glucosyl ceramide accumulates widely [230].

In order to examine the feasibility of this approach in vivo, mouse models of F508delCFTR and N370S GBA were tested following mCT treatment.

### 7.3. Cystic Fibrosis Animal Model

Although the F508delCFTR mouse does not fully reflect the lung pathology typical of human CF [231,232], intestinal epithelial chloride transport is ablated and residual F508delCFTR-dependent saliva production in this model, provides a convenient assay of systemic CFTR function [233,234], commonly used as an in vivo assay of potentially therapeutic drugs [235,236,237].

In preliminary experiments, we measured the saliva production in a single male F508delCFTR mouse and then treated the mouse i.p. with mCT (400 ng/25 gm mouse) every 48 h for 2 weeks. Our contention was that cell turnover in tissues in vivo could be less than in cells in culture, and that beneficial effect might therefore be accumulative. After the two-week treatment period, the CFTR dependent saliva production increased 5-fold. Following a subsequent two-week interval, we measured saliva production again and gave another two-week treatment course. The initial elevated saliva production was maintained and following the second course, production was increased a further threefold for a 15-fold increase overall.

**Figure 2:**
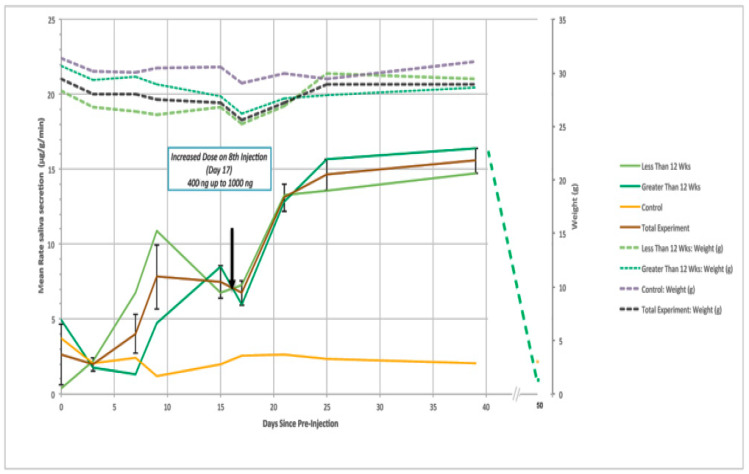
mCT restores F508delCFTR-dependent saliva production in the F508delCFTR mouse. Six Homozygous female F508delCFTR mice were injected i.v. with 400 ng (100 ng/mL blood) mCT every 48 h (from day 1) for two weeks. The dose was then increased to 1000 ng for a further three weeks, and CFTR dependent saliva secretion measured periodically (4 h post injection). Mice were left untreated for a further 11 days and saliva secretion measured. Three control mice were injected with saline and their saliva production did not change. No significant change in weight was observed for the treated mice, Saliva secretion in normal mice was measured as 6.7 µg/min/gm. After 40 days, the mice were left untreated for a further 10 days when the elevated saliva production returned to untreated levels. Importantly, no adverse effects were observed within the course of this study. These studies were performed under contract from ERAD Therapeutics by the Animal facilities at the Research Institute at the Hospital for Sick Children.

These encouraging preliminary results led to the studies reported in Figure 2, in which 1–3 week old female F508delCFTR mice were treated with repeated i.p. mCT doses/48 h and CFTR dependent saliva production monitored periodically during the treatment. The results clearly show that saliva production is markedly increased during the first 2 week treatment (by ~300%). There was a lag period which was greater for older mice, but all reached the same approximate increased value by day 15. After this time, the dose was increased, and saliva production was further increased during treatment (by ~100%) to a plateau value seven fold higher than control mice levels, which was maintained during the latter 2 weeks of this second 1000 ng mCT/48 h treatment period. Leaving the mice for a subsequent ten days without further treatment was sufficient for saliva production in the F508delCFTR mice to return to the pretreatment control baseline. No significant effect on weight or adverse behaviour (e.g., ruffled fur) was observed during the course of treatment.

These data confirm that repeated mCT administration is accumulative and that the F508delCFTR saliva production can be rescued by mCT to levels in marked excess of those found in wild type mice, indicating that this is a potent therapeutic approach to CF.

## 8. Gaucher Disease Animal Model

**Figure 3:**
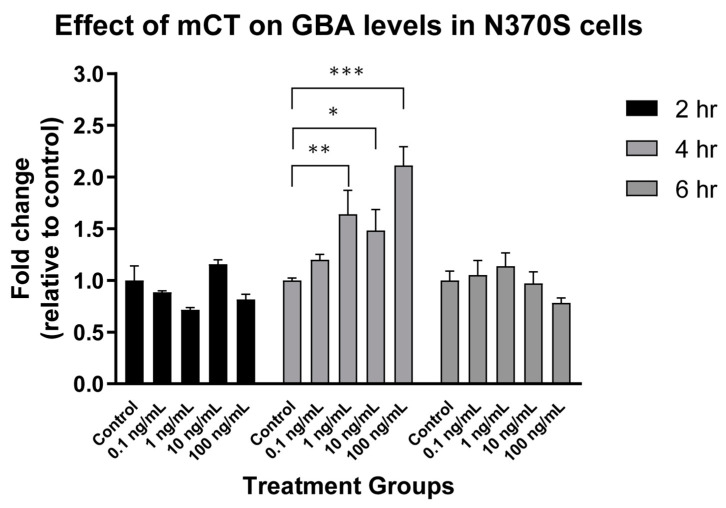
Effect of mCT on glucocerebrosidase A levels in N370 S Gaucher cells. N370S GBA Gaucher fibroblasts in 96 well microplate triplicates were treated with mCT as indicated and the expression level of GBA monitored by Western blot with MAB7410 using the quantitative Western Assay System (Protein Simple). These studies were carried out under contract by ERAD Therapeutics, in the laboratory of Dr Will Costain, NRC, Ottawa, ON, Canada. * *p* < 0.05, ** *p* < 0.01, *** *p* < 0.001.

In our original studies, we also showed that mCT was a viable approach to the treatment of N370S GBA Gaucher Disease cells in vitro [220]. This was subsequently verified as a function of both mCT dose and time (Figure 3). N370S GBA activity showed a dose dependent increase after 4 h treatment but this was lost after 6 h treatment. Thus, the rescue of N370S GBA was time dependent in cultured cells.

**Figure 4:**
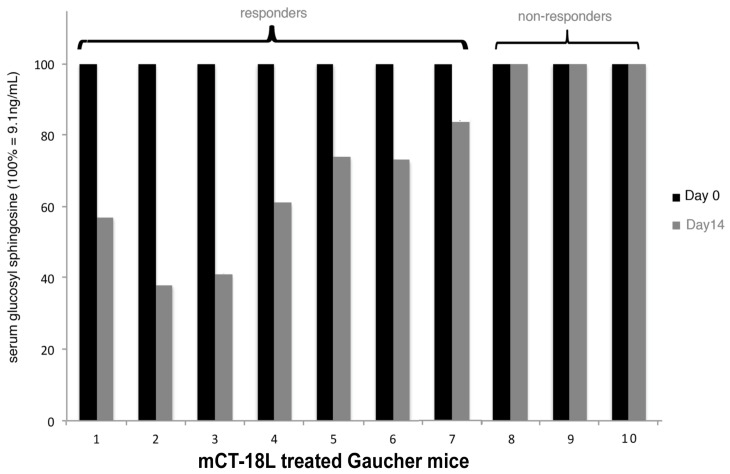
mCT reduces plasma glucosyl sphingosine in the N370S Gaucher mouse. N370S glucocerebrosidase Gaucher mice (25 gm) were injected with 400 ng mCT18L i.p. every 48 h for two weeks. Blood samples taken prior to (day 0) and after (day 14) the treatment period were then assayed for glucosyl sphingosine by mass spectrometry and the prior and after values compared. Thus, each mouse served as its own control. The mouse studies were performed under contract from ERAD Therapeutics, in the laboratory of Dr Lorne Clark, UBC and the (blinded) blood glucosyl ceramide mass spectrometric analyses were performed at the facilities at the Hospital for Sick Children.

The mouse model of N370S GBA Gaucher disease [238] was used to access the in vivo therapeutic potential of mCT (Figure 4). Following from the first cystic fibrosis mouse study, we reasoned that repeated treatment might be accumulative and therefore employed the same initial treatment regime. Serial assays of GBA activity were not feasible and therefore the direct measurement of accumulated GSL was measured before and after treatment. In Gaucher disease, serum glucosyl sphingosine (psychosine) accumulation has been shown to be a more informative monitor of clinical disease [239,240,241]. We therefore measured levels of glucosyl sphingosine in the serum of each mouse and compared that with the level after treatment in the same mouse. In our original cell culture studies, we added a hydrophobic C terminal hydrophobic peptide sequence (equivalent to a -100 or 50% -i.e.,an 18 or 9-polyleucine-stop transfer sequence) to the inactivated A subunit to attempt to increase the retrotranslocon transit time and thereby increase the efficacy of ERAD blockade. This indeed proved more effective in cell culture [220]. It was mCT-18L (mCT with an 18 leucine C terminal tail) which was used to treat the Gaucher mice. mCT-18L was more difficult to purify and, therefore, was not used in the CF studies.

In seven of the ten treated mice, a decrease in glucosyl sphingosine was observed ranging from 20% to 60%. This suggested that there was an additive effect on repeated mCT i.p. injection, as seen for CF mice, but unlike the CF mice, as yet unknown factors can prevent an mCT response in 30% of Gaucher mice.

Nevertheless, in sum these animal model studies of partial misfolding diseases show the in vivo feasibility of using the A subunit inactivated cholera holotoxoid as a therapeutic approach to genetic protein misfolding diseases in which the mutant protein retains partial wildtype function but is degraded by ERAD, due to the mutation-induced partial misfolding. Depending on the extent of ERAD involvement, there are 40–60 such diseases [242].

The finding that wildtype CFTR was increased via holotoxoid ERAD blockade [220], is consistent with significant wildtype CFTR misfolding, even under normal conditions [188]. Thus, if CFTR (or any normal protein similarly subject to ERAD) were in short supply, for whatever reason, holotoxoid ERAD blockade might prove an effective remedy.

## 9. Future Studies

The ERAD pathway is also co-opted by several pathogenic viruses [243] and inactivated holotoxin may prove effective against such infections also. The ER is a central and highly dynamic compartment in cell metabolism. The ability to target exogenous proteins, etc., to this compartment by (cleavable) coupling them to VT/CT B subunits, offers a largely untapped resource to modify a large array of local metabolic pathways. Since the ER membrane is continuous with the nuclear envelope, some years ago, we coupled a DNA binding element to VTB as a means to transport genes to the ER-> nucleus [244]. Such outside-the-box concepts still have a place in scientific progress.

## Data Availability

Not applicable.
